# Tenascin-W Is a Novel Stromal Marker in Biliary Tract Cancers

**DOI:** 10.3389/fimmu.2020.630139

**Published:** 2021-02-22

**Authors:** Ismaïl Hendaoui, Ahlem Lahmar, Luca Campo, Sihem Mebarki, Sandrine Bichet, Daniel Hess, Martin Degen, Nidhameddine Kchir, Leila Charrada-Ben Farhat, Rania Hefaiedh, Christian Ruiz, Luigi M. Terracciano, Richard P. Tucker, Lotfi Hendaoui, Ruth Chiquet-Ehrismann

**Affiliations:** ^1^ Friedrich Miescher Institute for Biomedical Research, Basel, Switzerland; ^2^ Department of Pathology, Mongi Slim University Hospital, La Marsa, Tunisia; ^3^ Medical School, University of Tunis El Manar, Tunis, Tunisia; ^4^ Laboratory for Oral Molecular Biology, Department of Orthodontics and Dentofacial Orthopedics, University of Bern, Bern, Switzerland; ^5^ Pathology Department, La Rabta University Hospital, Tunis, Tunisia; ^6^ Department of Diagnostic and Interventional Radiology, Mongi Slim University Hospital, La Marsa, Tunisia; ^7^ Department of Hepato-gastro-enterology, Mongi Slim University Hospital, Tunis, Tunisia; ^8^ Institute of Pathology, University Hospital Basel, Basel, Switzerland; ^9^ Department of Cell Biology and Human Anatomy, University of California at Davis, Davis, CA, United States; ^10^ Faculty of Science, University of Basel, Basel, Switzerland

**Keywords:** tenascin-W, tenascin-N, tenascin-C, gallbladder cancer, extracellular matrix, tumor stroma

## Abstract

Extrahepatic cancers of the biliary system are typically asymptomatic until after metastasis, which contributes to their poor prognosis. Here we examined intrahepatic cholangiocarcinomas (n = 8), carcinomas of perihilar bile ducts (n = 7), carcinomas of the gallbladder (n = 11) and hepatic metastasis from carcinomas of the gallbladder (n = 4) for the expression of the extracellular matrix glycoproteins tenascin-C and tenascin-W. Anti-tenascin-C and anti-tenascin-W immunoreactivity was found in all biliary tract tumors examined. Unlike tenascin-C, tenascin-W was not detected in normal hepatobiliary tissue. Tenascin-W was also expressed by the cholangiocarcinoma-derived cell line Huh-28. However, co-culture of Huh-28 cells with immortalized bone marrow-derived stromal cells was necessary for the formation and organization of tenascin-W fibrils *in vitro*. Our results indicate that tenascin-W may be a novel marker of hepatobiliary tumor stroma, and its absence from many normal tissues suggests that it may be a potential target for biotherapies.

## Introduction

Cancers originating in the epithelia of the biliary system are typically categorized as intrahepatic cholangiocarcinoma (ICC), carcinoma of perihilar bile ducts (CPHBD) or carcinoma of the gallbladder (CGB). While some early intrahepatic biliary system tumors have the potential to be treated effectively by radiotherapy, chemotherapy, local resection or liver transplantation, cancers of the extrahepatic biliary system have a particularly poor prognosis and limited options for therapy (1). In CGB the best outcomes require early diagnosis and resection, but extrahepatic lesions typically have asymptomatic development and most patients have metastatic disease by the time of surgery (2). The mean survival rate following CGB diagnosis is 6 months, and the 5-year survival rate is only 5% (3). CGB is more common in women than in men (4, 5) and while world-wide it is a rare cancer, it is relatively common in certain populations (2, 6–9). Some of these variations are likely related to regional rates of cholelithiasis, as 74–92% of patients with CGB have gallstones (10).

Tenascins are a family of extracellular matrix glycoproteins that share a common domain architecture (11). The best studied tenascin is the hexabrachion tenascin-C, which is required for normal embryonic development and reappears in the stroma of solid tumors (12, 13). Tenascin-C is abundant in the extracellular matrix of cancers of the breast, colon, pancreas, prostate, uterus, lung, skin, and brain, to name a few (14–16). *In vitro*, tenascin-C promotes the proliferation and migration of many tumor-derived cell lines (17) and there are reduced metastases in tenascin-C knockout mice and in mice lacking factors that promote the expression of tenascin-C (16). Tenascin-C may also play a role in creating an immune suppressive tumor stroma, thus contributing to tumor growth and metastasis (18). Tenascin-C has been proposed as a marker, immunotherapy target and indicator of prognosis in many kinds of cancer (16), but its use has been limited by its presence, albeit at lower levels, in many normal adult tissues (19).

Much less is known about tenascin-W, which is also known as tenascin-N (20, 21). It is particularly abundant in the embryo, especially at sites of osteogenesis (22). In the adult, it is found in certain stem cell niches together with tenascin-C (23) but except for the kidney and spleen its expression elsewhere in the adult is sparse (24, 25). Like tenascin-C, tenascin-W can promote cell migration *in vitro*, but it typically has little or no effect on cell proliferation (21). Tenascin-W is also found in the stroma of certain solid tumors. This was first reported in murine breast carcinomas (26) and later in human breast and colon cancer (27, 28), and glioblastomas (29). Studies with normal human tissue samples suggest that the expression of tenascin-W may be more restricted in the adult than tenascin-C and may therefore be a better marker of tumor stroma and a more appropriate target for biotherapies (19).

Here we used immunohistochemistry to study the expression of tenascin-C and tenascin-W in the stroma of ICC, CPHBD and CGB, and we characterized biliary tract cancer-derived cell lines for tenascin-W expression. We also studied the expression of tenascin-W in a wide array of normal human tissues. Our goals were to determine if tenascin-W represents a novel candidate for use as a biliary tract cancer stromal marker and if tenascin-W is a potential target for adjuvant therapy to fight cancers of the biliary system.

## Material and Methods

### Cases

This study was approved by the Mongi Slim University Hospital (MSUH) Committee on Medical Ethics (La Marsa, Tunisia) and the Ethikkommission Nordwest- und Zentralschweiz (Switzerland). All methods were performed according to these approved guidelines and regulations and are consistent with the editorial and publication policies of this journal. Only MSUH patients who underwent biopsy or surgery for diagnosis or treatment purposes were asked to participate by signing an informed consent form. None of the patients received radio and/or chemotherapy before biopsy or surgery. 17/18G percutaneous needle biopsies or surgical resection were fixed in Shandon™ Formal-Fixx™ 10% Neutral Buffered Formalin (Thermo Fisher Scientific, Runcorn, UK) for 24 h and then shipped in phosphate buffered saline (PBS) at 4°C to the Friedrich Miescher Institute for Biomedical Research (Basel, Switzerland) for processing. Upon reception, samples were incubated overnight in PBS with 30% sucrose for cryoprotection before being frozen at -80°C in Tissue-Tek OCT Compound (Sakura Finetek USA, Torrance, CA, USA) using acetone precooled with dry-ice. Histological diagnosis of each biopsy/resection was performed by at least one of two pathologists (A. L. and N. K.) according to the World Health Organization classification of tumors (30). Tumors were staged according to the Union for International Cancer Control 8^th^ TNM Classification of Malignant Tumors. Formalin-fixed and paraffin-embedded tissue samples were obtained from the Institute of Pathology of the University Hospital of Basel (Basel, Switzerland) and FDA standard human adult normal tissue frozen arrays were purchased from Biochain (Hayward, CA, USA).

### Immunohistochemistry

Both monoclonal mouse anti-human tenascin-C (B28-13) and anti-human tenascin-W (56O) antibodies were generated and characterized as described previously (27, 31). Immunohistochemistry experiments were performed with a Ventana Discovery Ultra instrument (Roche Diagnostics, Manheim, Germany). For fixed-frozen material, the procedure RUO Discovery Universal was used. Sections were first fixed with 4% formaldehyde for 12 min on-line and anti-tenascin-C B28-13 (1:10000) or anti-tenascin-W 56O (1:30) were applied manually and incubated for 1 h at 37°C. For anti-tenascin-W, a blocker (Discovery antibody block, Roche Diagnostics) was applied. To detect bound antibodies, a monoclonal rabbit-anti-mouse IgG (ab133469, Abcam, Cambridge, UK) was used as a linker (0.6 μg/ml) and applied for 20 min. Antibody-antigen complexes were detected with ImmPRESS anti-rabbit Ig (peroxidase) polymer reagent (MP-7401, Vector Laboratories, Burlingame, CA, USA) applied manually and incubated for 32 min. The ChromoMap DAB kit (Roche Diagnostics) was used for detection. Finally, slides were counterstained with Hematoxylin II and Bluing Reagent (Roche Diagnostics) for 8 min.

For formalin-fixed, paraffin-embedded material, the procedure RUO Discovery Universal was used with 40 min CC1 pre-treatment. Anti-tenascin-C B28-13 (1:5000) or anti-tenascin-W 56O (1:100) were applied manually and incubated for 1 h at 37°C or 3 h at room temperature, respectively. For anti-tenascin-C, secondary antibody (ImmPRESS reagent kit peroxidase anti-mouse Ig MP-7402, Vector Laboratories) was applied manually (200 µl) and incubated for 32 min at 37°C. For anti-tenascin-W, a blocker (Dako antibody diluent with background reducing component, S3022, Dako Agilent Pathology Solutions, Santa Clara, CA, USA) was applied together with the primary antibody. Detection of bound anti-tenascin-W antibody was achieved by incubating 20 min with anti-mouse HQ (Roche Diagnostics) and 24 min with anti-HQ HRP (Roche Diagnostics). Finally, for both anti-tenascin-C and anti-tenascin-W immunostainings, ChromoMap DAB kit (Roche Diagnostics) was used for the detection and slides were counterstained with Hematoxylin II and Bluing Reagent (Roche Diagnostics) for 8 min.

For controls, sections were processed as above but without primary antibodies. In addition, mouse IgG2A (Clone # 20102, R&D systems) and mouse IgG1 (Clone # 11711, R&D systems) were used as isotypic controls for anti-tenascin-C and anti-tenascin-W, respectively. Control slides were unlabeled.

### Cell Lines

Twenty human biliary tract cancer cell lines were collected from different sources. Huh-28 (ICC), HuCCT1 (ICC), OZ (CPHBD), NOZ (CGB) and OCUG-1 (CGB) cell lines were obtained from the Japanese Collection of Research Bioresources Cell Bank. TKKK (ICC), TFK-1 (CPHBD), TGBC1TKB (CGB), TGBC2 (CGB), TGBC14TKB (CGB), TGBC24TKB (CGB) and G-415 (CGB) cell lines were obtained from the RIKEN BioResource Center. SNU-1079 (ICC), SNU-245 (CPHBD), SNU-1196 (CPHBD) and SNU-308 (CGB) cell lines were obtained from the Korean Cell Line Bank. The Egi-1 (CPHBD) cell line was obtained from the Leibniz Institute DSMZ - German Collection of Microorganisms and Cell Cultures. All cell lines were cultured as recommended by their respective cell banks. SK-ChA-1 (CPHBD), Mz-ChA-1 (CGB), Mz-ChA-2 (CGB) were obtained from Prof. Alexander Knuth (University Hospital of Zürich, Zürich, Switzerland) (32) and cultured in RPMI 1640 with 10 mM HEPES, 2 mM L-Glutamine, 1X MEM non-essential amino acids (Thermo Fisher Scientific), 100 U/ml penicillin, 100 µg/ml of streptomycin, and 10% fetal bovine serum (FBS). Human BMSCs (196hT) immortalized with the hTERT/GFP system (33) were cultured as described previously (34). The control cell line 293/hTNW that stably expresses human tenascin-W (27) was cultured in DMEM with 0.25 µg/ml of G418, 1.5 µg/ml of puromycin, and 10% FBS.

### Cell Culture, Immunocytochemistry, and siRNA Transfection

For the co-culture assay, Huh-28 and 196hT cell lines were cultured in α-MEM plus 10% FBS, either alone or together at 1:1 ratio, with a total density of 27000 cells/cm². Cells were cultured in BD Falcon™ 8-well CultureSlides (BD Biosciences, Franklin Lakes, NJ, USA) pre-coated with 0.01% poly-L-lysine (Cultrex, Trevigen, Gaithersburg, MD, USA) for 2 h at 37°C. For the transwell assay, Huh-28 and 196hT cell lines were cultured in 12-well plates containing inserts separated by a polycarbonate membrane with 0.4 µm pores (Costar, Corning Amsterdam, Netherlands). Huh-28 or 196hT cells were either plated in the upper chamber (30,000 cells in 0.5 ml medium) or on 10-mm round glass coverslips pre-coated with fibronectin (50 µg/ml for 2 h at 37°C) and placed in the lower chamber (100,000 cells in 1.5 ml medium). Huh-28 or 196hT were cultured in the lower chamber either alone (with only medium in the upper chamber) or in co-culture (with 196hT or Huh-28, respectively, in the upper chamber). Cells were cultured in α-MEM plus 10% FBS. Only the cells from the lower chamber can be analyzed by immunofluorescence, thanks to their culture on the pre-coated glass coverslip. For both co-culture and transwell assays, after 4 days of culture without changing the medium, cells were fixed with 4% formaldehyde (Electron Microscopy Sciences, Hatfield, PA, USA) diluted in PBS for 10 min at room temperature, permeabilized for 5 min with 0.1% Triton X-100 (Sigma-Aldrich, St. Louis, MO, USA), washed twice with PBS, and blocked for 15 min with 3% bovine serum albumin (Sigma-Aldrich) in PBS. Cells were then incubated for 1 h with anti-tenascin-W (56O) diluted at 1:100. After 3 washes with PBS, cells were incubated for 1 h in the dark with goat anti-mouse antibody conjugated to Alexa Fluor^®^ 568 (Thermo Fisher Scientific) diluted at 1:1000. Finally, cell nuclei were counterstained with DAPI (Sigma-Aldrich) and slides were mounted with ProLong^®^ Diamond Antifade Mountant (Life Technologies, Waltham, MA, USA). Images were acquired with an Axio Imager Z1 microscope (Zeiss, Oberkochen, Germany).

For siRNA transfection, Huh-28 cells were seeded in 6-well plates with a density of 600,000 cells/well. The day after, cells were transfected with 25 pmol/well of different Silencer Select^®^ SiRNAs (Thermo Fischer Scientific) using Lipofectamine RNAiMAX Transfection Reagent (Thermo Fisher Scientific) following manufacturer’s instructions. SiRNA TNW 1 (sc34270), siRNA TNW 2 (sc34271) and siRNA TNW 3 (sc34272) were used to knockdown tenascin-W. SiRNA control 1 (Silencer^®^ Negative Control #1, ref 4390843) and siRNA control 2 (Silencer^®^ Negative Control #2, ref 4390846) were used as negative control. Cells were collected 3 days post-transfection and tenascin-W expression was analyzed by Western blot as described below.

### Conditioned Media Preparation

Huh-28, 196hT and 293/hTNW cells were cultured in medium plus FBS 10% (RPMI 1640, α-MEM and DMEM, respectively) until reaching approximately 90% confluency. Then, cells were starved in their respective medium without FBS and antibiotics for 6 days (Huh-28 and 196hT cell lines) or 3 days (293/hTNW cell line) before collecting their conditioned medium. Finally, cell debris were removed by centrifugation and the conditioned medium was concentrated 15X (for Western blots) or 30X (for mass spectrometry) *via* precipitation with 10% trichloroacetic acid, before being resuspended in 4X sample buffer (0.2M Tris-HCl, 145.56mM SDS, 20% glycerol, bromophenol blue) containing 0.1M DTT.

### Western Blotting

For cell lysates, cells were lysed in RIPA buffer (50 mM Tris HCl pH 7.4, 150 mM NaCl, 0.2% Na-deoxycholate, 25 mM Hepes, 5 mM MgCl_2_) containing protease inhibitors (Complete Mini, EDTA-free, Roche Diagnostics). Protein concentration was determined with a Bradford Assay (BradfordUltra, Expedeon, San Diego, CA, USA). Cell lysates or conditioned media were separated on 7% polyacrylamide gel under reducing conditions and blotted to polyvinyl-difluoride membranes (Thermo Fisher Scientific). Membranes were then stained with Ponceau S to control for equal protein loading and blotting efficiency. After blocking for 1 h at room-temperature with 5% Skim Milk Powder (Sigma-Aldrich) in PBS-Tween-20 0.1% (PBS-T), Blots were incubated overnight at 4°C with either anti-tenascin-W (56O) diluted at 1:1000, or anti-GAPDH (ab9485, Abcam, Cambridge, UK) diluted at 1:1000, as primary antibodies. After several washing steps with PBS-T, peroxidase-conjugated anti-mouse IgG (G21040, Life Technologies) or anti-rabbit IgG (G21234, Life Technologies) for 1 h at room temperature to detect anti-tenascin-W or anti-GAPDH, respectively. Signal from immunoblots was detected by enhanced chemiluminescence using SuperSignal™ West Dura Extended Duration Substrate (Thermo Fisher Scientific), and exposed to Super RX films (Fujifilm, Dielsdorf, Switzerland).

### RNA Isolation and Gene Expression Analysis by qRT-PCR

Total RNA was isolated by using QIAshredder and RNeasy Mini Kit (QIAGEN, Hilden, Germany/Venlo, Netherlands). RNA was reverse transcribed into cDNA using the High Capacity cDNA Reverse Transcription Kit (Life Technologies) with oligo-p(dT)15 (Roche) instead of the random primers provided in the kit. Quantitative RT-PCR assay was performed with 50 ng of cDNA from each cell line, using Platinium SYBR Green qPCR SuperMix-UDG with ROX (Invitrogen, Life Science, Carlsbad, CA, USA) on a StepOnePlus™ Real-Time PCR System (Life Technologies). Relative expression of human tenascin-W was calculated using the ΔΔCT method, normalizing values to human TBP (TATA-Box binding protein) within each sample. Primers for human tenascin-W (forward primer: 5’-ATGCCCTCACAGAAATTGACAG-3’ and reverse primer: 5’-TCTCTGGTCTCTTGGTCGTC-3’) and for human TBP (forward primer: 5’-TGCACAGGAGCCAAGAGTGAA-3’ and reverse primer: 5’-CACATCACAGCTCCCCACCA-3’) were tested for specificity and efficiency.

### Mass Spectrometry

Mass spectroscopy was used to determine the identity of the high molecular weight band recognized by anti-tenascin-W on Western blots of Huh-28 cell line. For the samples, 293/hTNW cell lysate and Huh-28 serum-free conditioned medium were prepared as described above. To analyze the samples by spectrometry, 50µl of 30X concentrated serum-free Huh-28 conditioned medium and 250µg of 293/hTNW cell lysate were separated on a 7% SDS-PAGE gel and stained with InstantBlue™ (Expedeon Inc.) in order to visualize the bands of interest. The protein bands were excised from the gel, reduced with 10mM TCEP, alkylated with 20mM iodoacetamide and cleaved with 0.1 µg porcine sequencing grade trypsin (Promega) in 25mM ammonium bicarbonate (pH 8.0) at 37°C for 16 h. The extracted peptides were analyzed by capillary liquid chromatography tandem mass spectrometry with an EASY-nLC 1000 using the two-column set up (Thermo Scientific). The peptides were loaded in 0.1% formic acid, 2% acetonitrile in H_2_O onto a peptide trap (Acclaim PepMap 100, 75um x 2cm, C18, 3um, 100Å) at a constant pressure of 800 bar. Then they were separated at a flow rate of 150 nl/min with a linear gradient of 2–6% buffer B (0.1% formic acid in acetonitrile) in buffer A (0.1% formic acid) for 3 min followed by an linear increase from 6–22% for 40 min, 22–28% for 9 min, 28–36% for 8 min, and 36–80% for 1 min. The column was finally washed for 12 min in 80% buffer B on a 50µm x 15cm ES801 C18, 2µm, 100Å column mounted on a DPV ion source (New Objective) connected to a Orbitrap Fusion (Thermo Scientific).

The data were acquired using 120000 resolution for the peptide measurements in the Orbitrap and a top T (3 s) method with HCD fragmentation for each precursor and fragment measurement in the LTQ. Mascot Distiller 2.5 and MASCOT 2.5 (Matrix Science, London, UK) searching the human subset of the UniProt version 2015_01 data base combined with known contaminants was used to identify the peptides. The enzyme specificity was set to trypsin allowing for up to three incomplete cleavage sites. Carbamidomethylation of cysteine (+57.0245) was set as a fixed modification, oxidation of methionine (+15.9949 Da) and acetylation of protein N-termini (+42.0106 Da) were set as variable modifications. Parent ion mass tolerance was set to 10 ppm and fragment ion mass tolerance to 0.6 Da. The decoy function in Mascot was used and the results were validated and the false discovery rate (FDR) was calculated with the program Scaffold Version 4.4 (Proteome Software, Portland, USA). The peptide threshold was set to an FDR of 0.1% and for proteins to 1%.

### Cell Sorting

Huh-28 and 196hT cell lines were cultured in α-MEM plus 10% FBS, either alone or together at a ratio of 1:1, with a total density of 27,000 cells/cm². After 4 days of culture, using the ability of 196hT cells to express GFP, cells from both the co-culture and the monocultures were sorted with a BD Influx cell sorter (BD Biosciences) and BD FACS™ Sortware sorter software (Version 1.01.654). The sort was performed with a 100-mm nozzle tip, at a sheath pressure of 19.0 psi, and a frequency of 28.90 kHz. The following gates were set: gate 1 was forward scatter FSC-Area against side scatter SSC-Area, gate 2 was FSC-Width against FSC-Height, gate 3 was SSC-Width against SSC-Height, and gate 4 was 530/40 (488 nm) against 710/50 (561 nm).

## Results

**Figure 1:**
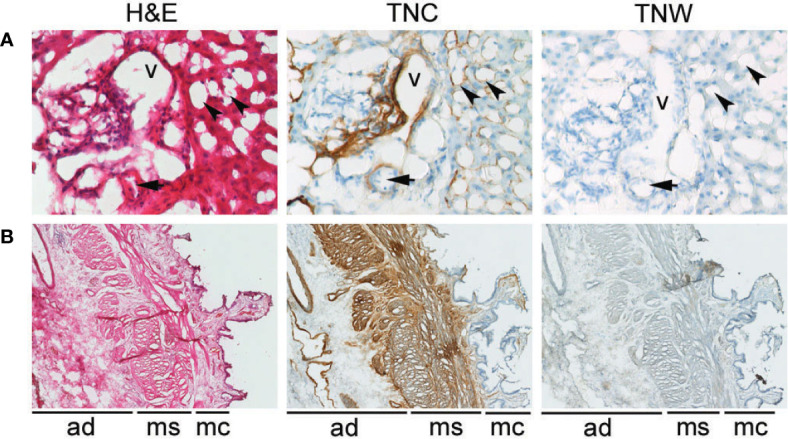
Anti-tenascin-C (TNC) and anti-tenascin-W (TNW) immunohistochemistry of hepatobiliary tissues. **(A)** TNC immunostaining is observed around sinusoids (arrowheads) and around a vein (v) and an artery (arrow) in portal tract sections of a normal adult liver. An adjacent section is not labeled with TNW immunohistochemistry (400X). **(B)** TNC immunohistochemistry of a non-tumoral adult gallbladder (cholecystitis) shows labeling around vessels in the adventitia (ad), as well as around bundles of smooth muscle in the muscularis layer (ms). There is also patchy immunoreactivity in the mucosa (mc). In contrast, TNW does not immunolabel gallbladder tissue (40X). A third section in each series was stained with hematoxylin and eosin (H&E).

**Figure 2:**
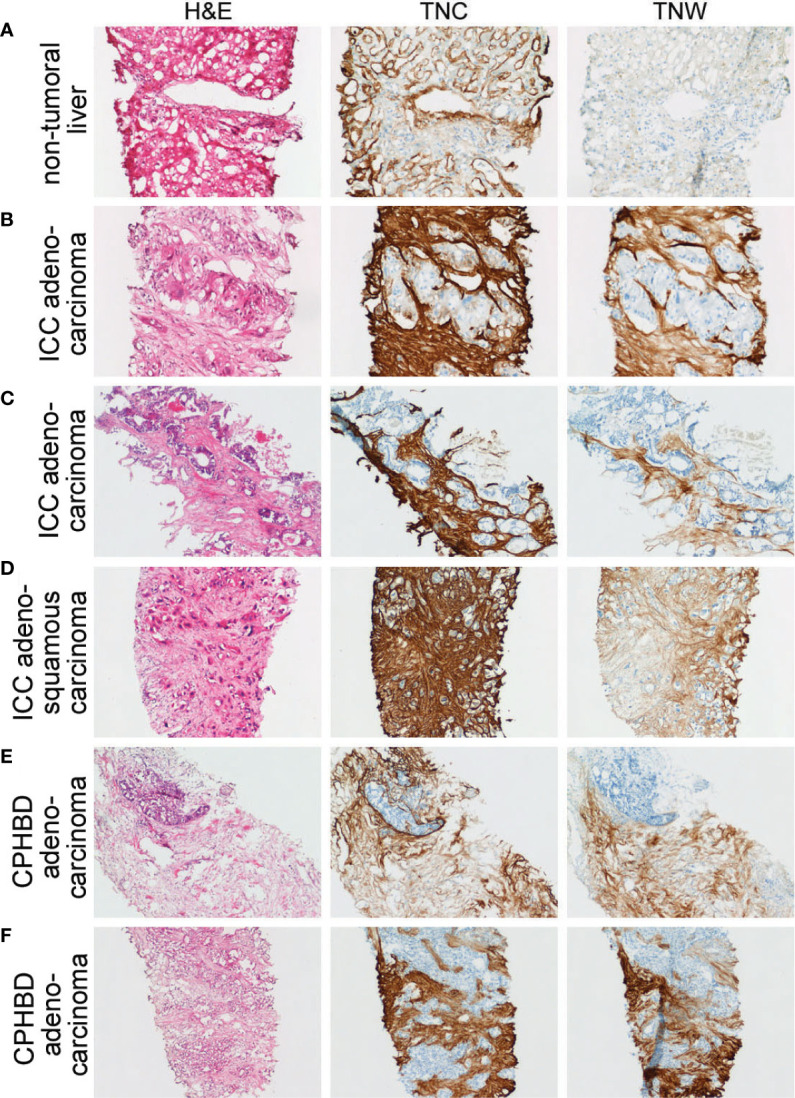
Adjacent sections through needle biopsies of non-tumoral liver and various intrahepatic cholangiocarcinomas and carcinomas of perihilar bile ducts immunostained with anti-tenascin-C (TNC) and anti-tenascin-W (TNW). **(A)** Non-tumoral liver (patient ID 5) is immunostained with TNC, but not TNW (200X). **(B–D)** Immunohistochemical examination of intrahepatic cholangiocarcinomas (ICC). Both TNC and TNW label the stroma of a moderately differentiated adenocarcinoma **(B)** strongly (patient ID 5; 200X). Antibodies to both tenascin-C and tenascin-W label the stroma of a well differentiated adenocarcinoma (Patient ID 1; **C**; 100X) and an adenosquamous carcinoma (Patient ID 8; **D**; 100X). **(E, F)** Immunolabeling of carcinomas of perihilar bile ducts (CPHBD) with TNC and TNW. The stroma of a moderately differentiated adenocarcinoma (Patient ID 11; **E**; 100X) and a well differentiated adenocarcinoma (Patient ID 13; **F**; 100X) show expression of both tenascin-C and tenascin-W. A third section in each series was stained with hematoxylin and eosin (H&E).

**Figure 3:**
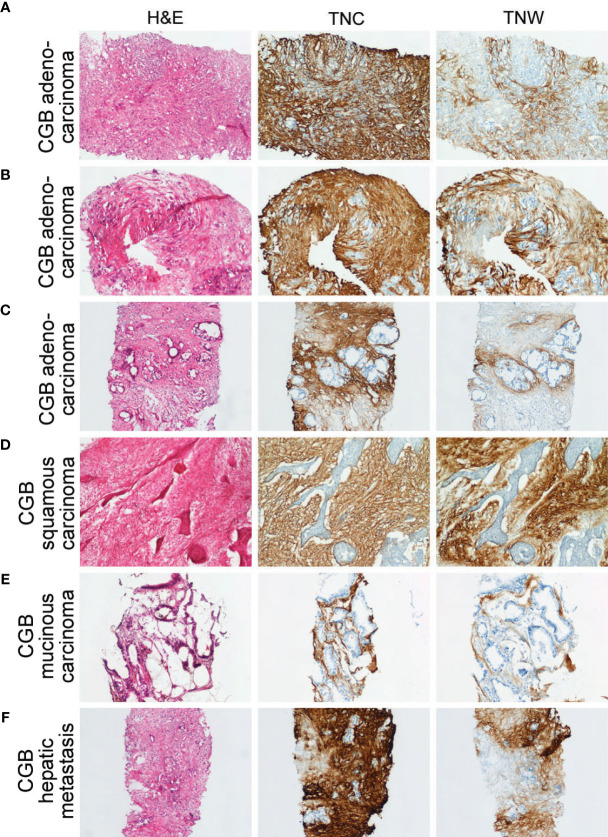
Sections through needle biopsies of carcinomas of the gallbladder and a hepatic metastasis from a carcinoma of the gallbladder stained with hematoxylin or immunostained with anti-tenascin-C (TNC) or anti-tenascin-W (TNW). **(A–E)** Immunostaining of carcinomas of the gallbladder (CGB). The stroma of a poorly differentiated adenocarcinoma (Patient ID 18; **A**), a moderately differentiated adenocarcinoma (Patient ID 17; **B**) and a well differentiated adenocarcinoma (Patient ID 26; **C**) are immunolabeled with both TNC and TNW (100X). Both TNC and TNW label the stroma of a squamous carcinoma (Patient ID 19; **D**; 100X) and a mucinous carcinoma (Patient ID 22; **E**; 100X). **(F)** A representative section through a needle biopsy of a hepatic metastasis from a carcinoma of the gallbladder (Patient ID 27) shows strong TNC and TNW immunolabeling in the tumor stroma (100X). A third section in each series was stained with hematoxylin and eosin (H&E).

In all, 30 tumors from patients with biliary tract cancers ranging in age from 29–79 years old were processed for histopathological examination as well as anti-tenascin-C and anti-tenascin-W immunohistochemistry. The results were compared with normal liver (all negative for HIV, hepatitis B, and hepatitis C), non-tumoral liver from a patient with ICC, and tissue from non-tumoral gallbladders that were removed due to cholecystitis. The results are summarized in **Supplementary Table 1** and **Table 1** and are illustrated in **Figures 1**–**3**.

**Table 1:** Summary of tenascin immunostaining of normal liver, non-tumoral gallbladder, and hepatobiliary cancers.

Tissue	Anti-TNC				Anti-TNW*			
	-	+	++	+++	-	+	++	+++
Liver (n = 13)**	0/13	5/13	7/13	1/13	13/13	0/13	0/13	0/13
Gallbladder (n = 6)^†^	0/6	1/6	2/6	3/6	5/6	1/6^¶^	0/6	0/6
ICC (n = 8)^‡^	0/8	0/8	0/8	8/8	0/8	1/8	2/8	5/8
CPHBD (n = 7)^§^	0/7	0/7	1/7	6/7	0/7	2/7	2/7	3/7
CGB (n = 11)^‖^	0/11	0/11	0/11	11/11	0/11	1/11	3/11	7/11
CGB liver metastasis (n = 4)	0/4	0/4	0/4	4/4	0/4	0/4	2/4	2/4

*Intensity of anti-tenascin-C (TNC) and anti-tenascin-W (TNW) immunostaining: -, absent; +, weak; ++, moderate; +++, high.

**Histologically normal liver, negative for HIV, hepatitis B, and hepatitis C.

^†^From cholecystectomy due to cholecystitis.

^‡^Intrahepatic cholangiocarcinoma.

^§^Carcinoma of perihilar bile ducts.

^‖^Carcinoma of the gallbladder.

^¶^Focal positivity.

Tenascin-C immunoreactivity is seen in hepatic sinusoids and around blood vessels (vein and artery) in the portal tract of normal liver tissue (**Figure 1A**). Conversely, anti-tenascin-W does not immunostain normal hepatic tissue (**Figure 1A**). Similarly, tenascin-C immunoreactivity is seen surrounding blood vessels in the adventitia of a non-tumoral gallbladder removed from a patient with cholecystitis, around bundles of smooth muscle in the muscularis externa, and in the lamina propria of the mucosa (**Figure 1B**). Tenascin-W immunoreactivity is not detected in the non-tumoral gallbladder (**Figure 1B**) and was not detected in tissues isolated from 5 of the 6 non-tumoral gallbladders examined (**Table 1**). The anti-tenascin-W immunostaining observed in one non-tumoral gallbladder was weak (+) and only present in a single focal fibrotic area (**Table 1**). The presence of tenascin-C immunoreactivity in all the non-tumoral tissues examined, and the general absence of tenascin-W immunoreactivity in adjacent sections, led us to examine a broad array of normal human tissues with the tenascin-W antibody (**Supplementary Figure 1**). As reported by others studying tenascin-W in mice (24, 25), the anti-tenascin-W immunostains extracellular matrix in normal adult human spleen and kidney. Tenascin-W immunoreactivity is also seen in the female reproductive system (uterus, uterine tubes and ovaries) as well as in certain glands (adrenal gland, pituitary gland, thyroid and prostate). Tenascin-W immunoreactivity is not seen in heart, lung or central nervous system samples, and it is absent or weak in tissues from the gastrointestinal system (esophagus, stomach, pancreas or colon). Consistent with our findings (**Figure 1A**), tenascin-W immunoreactivity is absent from three additional normal liver samples that were part of the array.

There is strong anti-tenascin-C immunoreactivity around sinusoids in the non-tumoral region of a liver sample taken from a patient with ICC (**Figure 2A**). As in the non-tumoral liver, there is no tenascin-W immunoreactivity in this sample. However, both antibodies immunostain the stroma surrounding the ICC itself (**Figure 2B**). Both tenascin-C and tenascin-W antibodies labeled the stroma of each ICC (n = 8) and CPHBD (n = 7) examined (**Figure 2** and **Supplementary Table 1**). The intensity of the anti-tenascin-W immunoreactivity in ICC and CPHBD is variable, but no clear correlations could be made between the intensity of the immunostaining and the tumor grade (**Supplementary Table 1**). While most of the ICC and CPHBD examined were well or moderately differentiated adenocarcinomas (n = 13), the tenascin-W antibody also immunolabeled the stroma of a single squamous cell carcinoma (**Supplementary Table 1**) and a single adenosquamous carcinoma (**Figure 2D**).

In all, 11 cases of CGB, as well as 4 independent CGB metastases to liver, were examined with immunohistochemistry (**Figure 3** and **Supplementary Table 1**). Anti-tenascin-C and anti-tenascin-W both immunolabel the stroma of poorly (n = 3), moderately (n = 4) and well differentiated adenocarcinomas (n = 2), as well as the stroma of a single squamous cell carcinoma (**Figure 3D**) and a single mucinous carcinoma (**Figure 3E**). There is also tenascin-C and tenascin-W immunoreactivity in the stroma of CGB hepatic metastases (**Figure 3F**). As with the ICC and CPHBD immunostaining, anti-tenascin-W immunolabels the stroma of various tumors with different intensities, but no clear correlations could be made with the intensity of the immunostaining and the tumor grade (**Table 1**).

To maximize anti-tenascin immunostaining, the ICC, CPHBD and CGB samples shown here were processed using cryopreservation and sectioning. However, following antigen retrieval it was also possible to use the antibodies to immunostain formalin-fixed and paraffin-embedded samples of CPHBD, ICC and CGB (**Supplementary Figure 2**).

**Figure 4:**
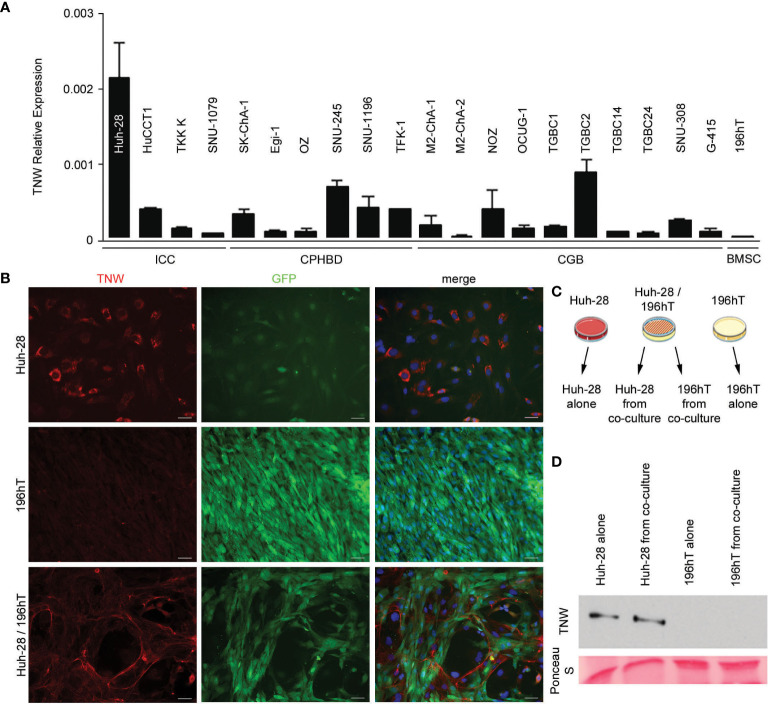
Expression of tenascin-W by cell lines derived from biliary tract tumors. **(A)** Twenty cell lines were analyzed for tenascin-W expression using qRT-PCR. The results were plotted as expression relative to that of the housekeeping gene hTBP. Four cells lines were derived from intrahepatic cholangiocarcinomas (ICC), 6 were from carcinoma of perihilar bile ducts (CPHBD), and 10 were from carcinoma of the gallbladder (CGB). For comparison, qRT-PCR expression of tenascin-W by a bone marrow stromal cell-derived cell line (196hT) is shown. The highest level of tenascin-W expression was seen in the ICC-derived cell line Huh-28; **(B)** Immunocytochemistry with anti-tenascin-W (TNW) reveals intracellular labeling of Huh-28 cells, but not 196hT cells. The 196hT cells, but not the Huh-28 cells, express GFP. The merged images include a DAPI nuclear counterstain. When the two cell lines are co-cultured, tenascin-W-positive fibrils surround nests of Huh-28 cells, mimicking the arrangement of tenascin-W in the stroma of ICCs. Scale bars = 50 μm. **(C)** The method used for separating Huh-28 cells from GFP-positive 196hT cells following co-culture is shown schematically. Illustration was created using Servier Medical Art Powerpoint Image Bank (Servier, https://smart.servier.com/). **(D)** Tenascin-W can be detected by immunoblotting with 30µg of cell lysates of Huh-28 cells and Huh-28 cells following co-culture with 196hT cells. Tenascin-W is not detectable in the lysates of 196hT cells or 196hT cells following co-culture. Ponceau S was used as a loading control.

To determine the potential cellular origins of tenascin-W in biliary tract cancers and to facilitate future studies of the role of tenascin-W in these cancers, 20 different human biliary tract cell lines were examined for tenascin-W RNA expression by qRT-PCR. While many of the cell lines had low to negligible levels of expression, ICC-derived Huh-28 cells expressed significant levels of tenascin-W (**Figure 4A**). This was confirmed by western blotting of Huh-28 cell lysates and 15X concentrated Huh-28 cell conditioned medium (**Supplementary Figure 3**), and by western blotting of lysates of Huh-28 cells before and after transfection with tenascin-W-specific siRNAs (**Supplementary Figure 4**) and by mass spectrometry (**Supplementary Figure 5**). Immunocytochemistry with the anti-tenascin-W shows bright intracellular staining of cultured Huh-28 cells, but no tenascin-W-positive fibrils were observed around cells. This leads us to speculate that Huh-28 cells secrete soluble tenascin-W but are not able to produce and organize tenascin-W-positive fibrils in the extracellular matrix, as observed in the tumor stroma of ICC, CPHBD and CGB patients (**Figure 4B**). Since bone marrow-derived stromal cells (BMSCs) represent a significant cellular source of cancer-associated fibroblasts that support tumor cell growth (35), we decided to perform co-culture between Huh-28 and the BMSC cell line 196hT in order to mimic a tumor stroma microenvironment. While 196hT cells do not express tenascin-W when cultured alone (**Figures 4A, B**), the tenascin-W labeled by immunocytochemistry in co-cultures of Huh-28 with 196hT cells is organized in fibrils surrounding nests of Huh-28 cells, mimicking the appearance of tenascin-W in the stroma surrounding nests of tumor cells in ICC. Similar results were observed with co-cultures of 196hT cells and other biliary tract cancer cell lines that express lower levels of tenascin-W: the CPHBD cell line TFK-1 and the CGB cell line G-415 (not shown). Since the 196hT cells were genetically engineered to express GFP (36), it was possible to sort the cells after co-culture and examine cell-type specific lysates for the expression of tenascin-W by western blotting (**Figure 4C**). Huh-28 cell lysates following co-culture had readily detectable tenascin-W, but 196hT cells did not (**Figure 4D**, **Supplementary Figure 6**), indicating that the source of the tenascin-W fibrils seen in the co-cultures is the Huh-28 cells and not the 196hT cells. To determine if physical contact between the cells or a secreted factor leads to the appearance of the fibrillar anti-tenascin-W immunostaining in the co-cultures, the two cells types were cultured in transwell chambers, where upper and lower chambers are separated by a porous polycarbonate membrane that allows the diffusion of soluble factors, but not the transmigration of cells. If 196hT cells are in the upper chamber, tenascin-W-positive fibrils are seen around Huh-28 cells in the lower chamber, in addition to the tenascin-W cytoplasmic localization observed in Huh-28 cells (**Supplementary Figure 7**). If Huh-28 cells are in the upper chamber, tenascin-W-positive fibrils are seen in 196hT cells in the lower chamber. However, no tenascin-W immunostaining is detected in the cytoplasm of 196hT cells. Therefore, soluble tenascin-W secreted by Huh-28 cells in the upper chamber becomes associated with fibrils when it enters into contact in the lower chamber with a soluble factor secreted by 196hT cells. These results suggest that a secreted factor in the tumor microenvironment, mimicked by co-cultures of Huh-28 and 196hT cells *in vitro*, is required for the production and assembly of tenascin-W-positive fibrils in the tumor stroma, and that the cancer cells themselves can be/are the source of tenascin-W, at least in ICC.

## Discussion

In the current study, tenascin-C immunoreactivity was observed in the stroma of all biliary tract tumors examined. Its presence in ICC is consistent with the reports of others who studied animal models (33) and intrahepatic tumors of biliary origin in humans (37–39). Antibodies to tenascin-C have been used as an ICC stromal marker in at least one animal study (40), and in an effort to identify combinations of antibodies that could be used for prognosis, a combination of high levels of tenascin-C immunoreactivity and low levels of osteopontin immunostaining was found to correlate with low post-surgery survival rates in patients with ICC (41). Our study appears to be the first to report tenascin-C immunoreactivity in the stroma of CPHBD and CGB, though one report has described elevated levels of tenascin-C in the serum of a patient with metastatic CGB (42). Thus, CPHBD and CGB can be added to the growing list of solid tumors in which tenascin-C expression has been well documented (14–17). Here, we report anti-tenascin-C immunostaining in normal liver around sinusoids, but also around veins and arteries in the portal tract, as was shown previously (43–45).

Tenascin-C exists in multiple isoforms, and different isoforms are found in different tumors (12). While this complicates the characterization of tenascin-C in tumor stroma, it has led to the development of prospective isoform-specific tumor therapies (12). The monoclonal antibody to tenascin-C used in this study (B28-13) recognizes an epitope common to all tenascin-C isoforms (19, 31). Less is known about the possible isoforms of tenascin-W, but to date immunoblots indicate that only a single form is expressed in human tumors (19, 29). Interestingly, the tenascin-W made by the ICC-derived cell line Huh-28 runs on SDS-PAGE with a higher apparent molecular weight than expected (**Supplementary Figure 4**). While this may be the result of multimerization, it may also represent a tumor-specific form or modification of the protein. To unambiguously identify the higher molecular weight band as tenascin-W we analyzed the tryptic peptides from this region by tandem mass spectrometry and compared them to the lower molecular weight band of tenascin-W derived from 293/hTNW cells (**Supplementary Figure 5**). Seven tenascin-W peptides could be identified with good MASCOT scores and a false discovery rate of 0.1%. They were manually evaluated and had similar retention times to the peptides derived from 293/hTNW cells. They are distributed over a wide range of the tenascin-W sequence.

The anti-tenascin-W used in the current study also immunostained the stroma of all the biliary tract tumors examined. However, in contrast to anti-tenascin-C, anti-tenascin-W did not immunostain normal liver, non-tumoral liver from 3 patients with ICC, or tissues from 5 of the 6 gallbladders removed from patients with cholecystitis. This makes tenascin-W a potential candidate for identifying stromal areas associated with biliary tract tumors and a potential target for biotherapies. Similar conclusions were drawn by others who found that tenascin-W immunoreactivity was associated with glioblastomas, astrocytomas and oligodendrogliomas, but was absent from normal brain tissue (29). Tenascin-W is also found in colorectal cancer, but not in normal colon tissue (28). For a more comprehensive view of tenascin-W expression in normal human tissues, we surveyed additional normal adult human tissues using anti-tenascin-W-based immunohistochemistry and found that many, but not all, lacked tenascin-W expression. Some normal glands (e.g., prostate) and the female reproductive system (e.g., the uterus and ovaries) displayed high levels of tenascin-W expression. This would need to be considered when developing systemic therapies or using tenascin-W as a potential marker of tumor stroma in these regions. Tenascin-W is elevated in the serum of patients with breast and colorectal cancer (28). It would be interesting to see if tenascin-W is a potential serum marker for biliary tract cancers as well, as reliable serum markers for these cancers have yet to be developed (46).

What is the origin of the tenascin-W found in the stroma of biliary tract tumors? We were unable to perform *in situ* hybridization with probes for tenascin-W transcripts on the tumor samples, as the fixation protocol was optimized for immunohistochemistry. However, the expression of tenascin-W in cell lines derived from ICC (Huh-28), CGB (TGCB2) and CPHBD (SNU245) is evidence that the cancer cells themselves may express some of the tenascin-W found in the tumor stroma (**Figure 4A**). However, our results do not rule out possible origins from cancer associated fibroblasts or endothelial cells. Futures studies should address this question.

Most *in vitro* studies of tenascin-W expression have involved primary cultures of embryonic osteoblasts as few cell lines have been identified that express tenascin-W (47, 48). Here, we found that tenascin-W transcripts are relatively high in Huh-28 cells, and tenascin-W can be detected in cell lysates and conditioned medium of Huh-28 cells (**Supplementary Figure 3**). Thus, Huh-28 cells are the first cell line identified that express tenascin-W without the addition of exogenous factors and may prove useful in future studies to elucidate tenascin-W function. The Huh-28 cell line was derived from an ICC removed with the left lobe of the liver from a 37-year-old woman in 1984 (49). They are large, spindle-shaped or polygonal cells that proliferate slowly under typical culture conditions and fail to form tumors when injected into nude mice (49). A future study could involve co-injection of BMSCs with the Huh-28 cells to see if this results in tumor formation. Because they share properties with primary cultures of cholangiocarcinoma cells, Huh-28 cells have also been used as an *in vitro* model for studying the regulation of ICC proliferation. Like primary ICC cells, Huh-28 cells express estrogen receptors and their serum-induced proliferation is inhibited with tamoxifen (50). They also proliferate in response to IGF-1 (50) and TGF-beta (51). Huh-28 cells also express relatively high levels of miR-24, a small non-coding RNA associated with oncogenesis (52). When co-cultured with immortalized BMSCs, we show here that Huh-28 cells form nests that are surrounded by fibrils that are immunostained with anti-tenascin-W, mimicking the arrangement of ICC cells surrounded by tenascin-W stroma *in situ*. This may prove to be a good model for future studies of ICC growth and the development of anti-ICC therapies. It is also important to note that as we were unable to acquire phase contrast images of the co-cultured cells, it is difficult to determine if the tenascin-W-positive fibrils were on the cell surface or were part of a broader fibrillar system. Future studies should also be directed at studying the effects of exogenous tenascin-W on Huh-28 cell proliferation and behavior, as was done with breast cancer cell lines (21).

The C-terminal fibrinogen-related domain of tenascin-W, like that of tenascin-C, can activate Toll-like receptor 4-mediated inflammatory responses (53). Moreover, tenascin-C was recently shown to contribute to immune suppression in an oral squamous cell carcinoma model (18). Future studies should be directed to determine if tenascin-W, which shares a number of features with tenascin-C, plays a similar role in tumor stroma, or if the presence of tenascin-W during cholelithiasis could contribute to tumor progression.

The extracellular matrix glycoprotein tenascin-W is found in the stroma of ICC, CPHBD and CGB. It is not expressed in normal liver or most cholecystitic gallbladders. Thus, tenascin-W is potentially a novel stromal marker for biliary tract cancers and may prove useful in the development of biotherapies.

## Data Availability Statement

The original contributions presented in the study are included in the article/**Supplementary Material**. Further inquiries can be directed to the corresponding author.

## Ethics Statement

The studies involving human participants were reviewed and approved by Mongi Slim University Hospital (MSUH) Committee on Medical Ethics (La Marsa, Tunisia) and the Ethikkommission Nordwest- und Zentralschweiz (Switzerland). The patients/participants provided their written informed consent to participate in this study.

## Author Contributions

Study concept and design: IH, RC-E and LH. Data acquisition and analysis: IH, AL, LC, SB, NK, DH, and SM. Data interpretation and discussion: IH, RC-E, RT, AL, LC, MD, LT, LH, and CR. Patient recruitment: LH and RH. Clinical diagnosis: LH and LC-B. Taking biopsies: LH. Manuscript writing: RT, IH, and LH. Critical revision of the manuscript for important intellectual content: LT and MD. All authors contributed to the article and approved the submitted version.

## Funding

This work was supported by funds from the Swiss National Science Foundation (31003A_156740) to RC-E.

## Conflict of Interest

Some of the results showed herein have been used in the patent “Tenascin-W and biliary tract cancers” (WO2017072669).

The authors declare that the research was conducted in the absence of any commercial or financial relationships that could be construed as a potential conflict of interest.
