# Supplementary material for: Tenascin-W Is a Novel Stromal Marker in Biliary Tract Cancers
Source: Front Immunol. 2021 Feb 22;11:630139. doi: 10.3389/fimmu.2020.630139 (PMC7937617; doi:10.3389/fimmu.2020.630139)
Supplement: Supplementary file 1 [file DataSheet_1.docx]

Supplementary Material: Figures

# Supplementary Figures


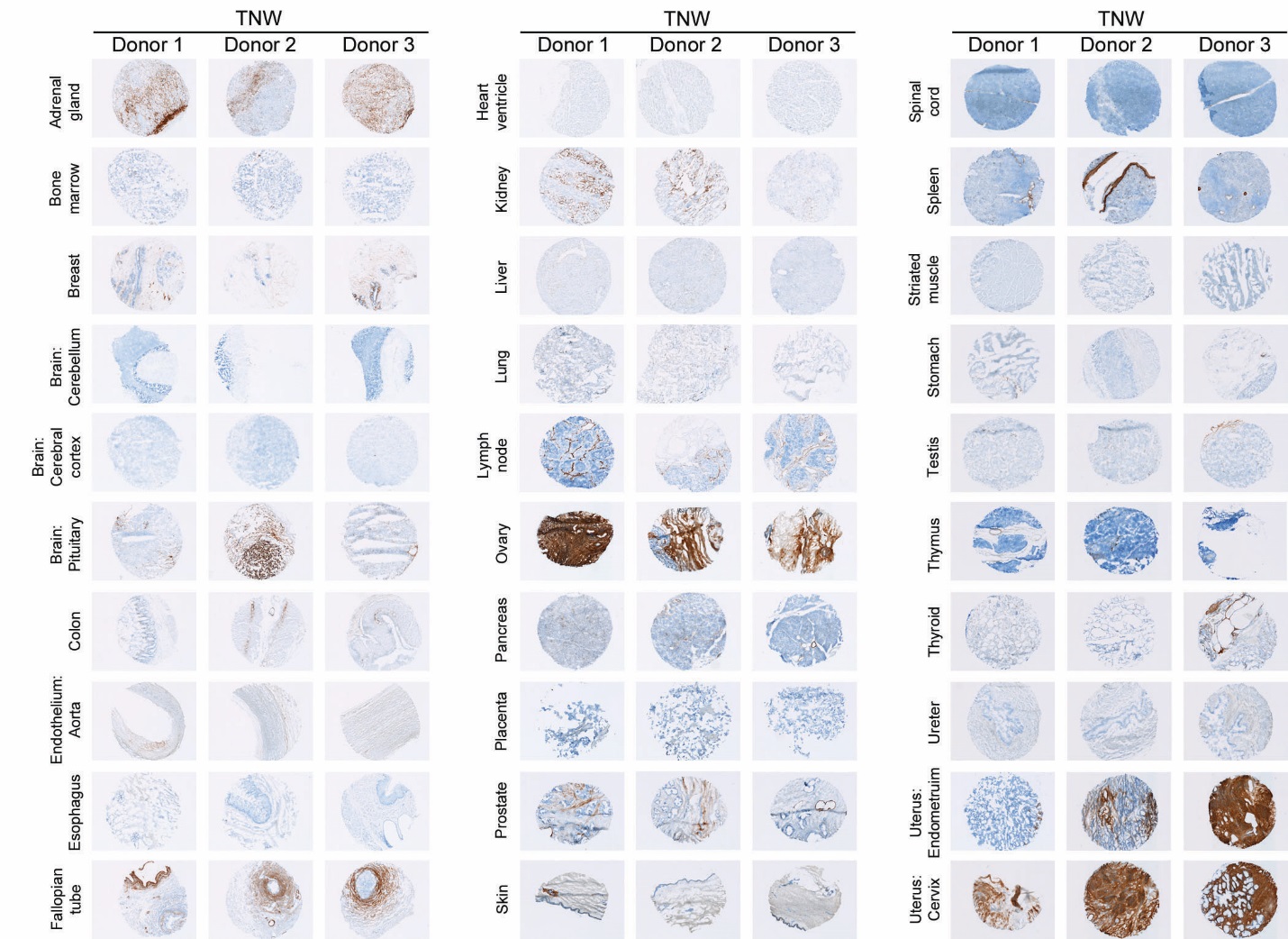


**Supplementary Figure 1** Immunohistochemical localization of tenascin-W (TNW) in an FDA standard human adult normal tissue array. For each organ, 3 different donors were used. TNW is detected in the extracellular matrix of normal adult human spleen, kidney, female reproductive system (uterus, uterine tubes and ovaries) and certain glands (adrenal gland, pituitary gland, thyroid and prostate), but not in normal adult human liver.

**Supplementary Figure 2** Anti-tenascin-C (TNC) and anti-tenascin-W (TNW) immunostaining of formalin-fixed and paraffin-embedded sections of biliary tract cancers. (**A,B**) Both TNC and TNW immunolabel the stroma of carcinomas of the perihilar bile ducts. (**C**) Tenascin-C and tenascin-W are both expressed in the stroma of carcinoma of the gallbladder. (**D**) TNC and TNW immunohistochemistry of an intrahepatic cholangiocarcinoma. Adjacent sections are stained with hematoxylin and eosin (H&E).


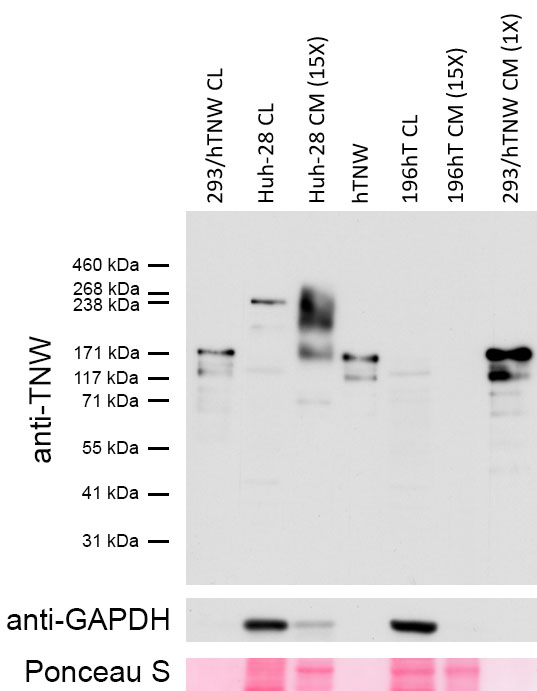


**Supplementary Figure 3** Immunoblot of cell lysates (CL) and conditioned medium (CM) from a control cell line stably transfected with human tenascin-W (293/hTNW), Huh-28 cells and 196hT cells. Cell lysates from 293/hTNW (1 µg), Huh-28 (50 µg) and 196hT (50 µg), and CM from 293/hTNW (1X, 0.5 µl), Huh-28 (15X, 40 µl) and 196hT (15X, 40µl) were subjected to electrophoresis and immunoblotted with anti-TNW. The center lane (hTNW) was loaded with 5 ng of recombinant human tenascin-W. Loading controls included anti-GAPDH and Ponceau S. Huh-28 cells secrete tenascin-W into the culture medium.


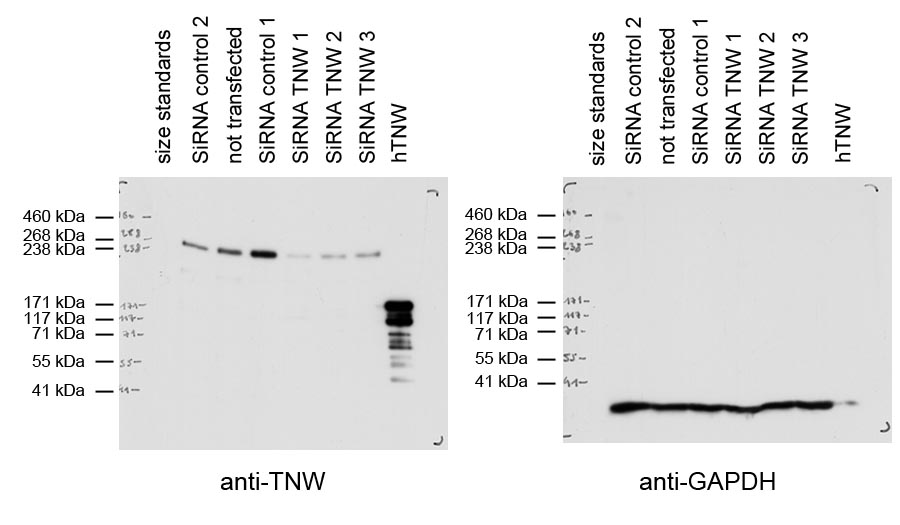


**Supplementary Figure 4** Immunoblots of cell lysates from the ICC-derived cell line Huh-28 (first 6 lanes) incubated with anti-tenascin-W (TNW; left) or anti-GAPDH (right) as a loading control. Recombinant human TNW (10 ng) is also shown as control (lane labeled hTNW). The band from the Huh-28 cell lysates recognized by anti-TNW runs at two to three times the apparent molecular weight of the control hTNW. To determine if this band is a form of TNW, Huh-28 cells were subjected to siRNA knockdown of TNW expression. The high molecular weight band is prominent in the Huh-28 cell lysates that were not transfected as well as in two independent siRNA controls (Silencer® Negative Control #1 and Silencer® Negative Control #2; 40 µg protein/lane). However, the band is only faintly labelled in Huh-28 cell lysates from cells transfected with three different TNW-specific siRNA constructs (40 µg protein/lane).

**A**

**
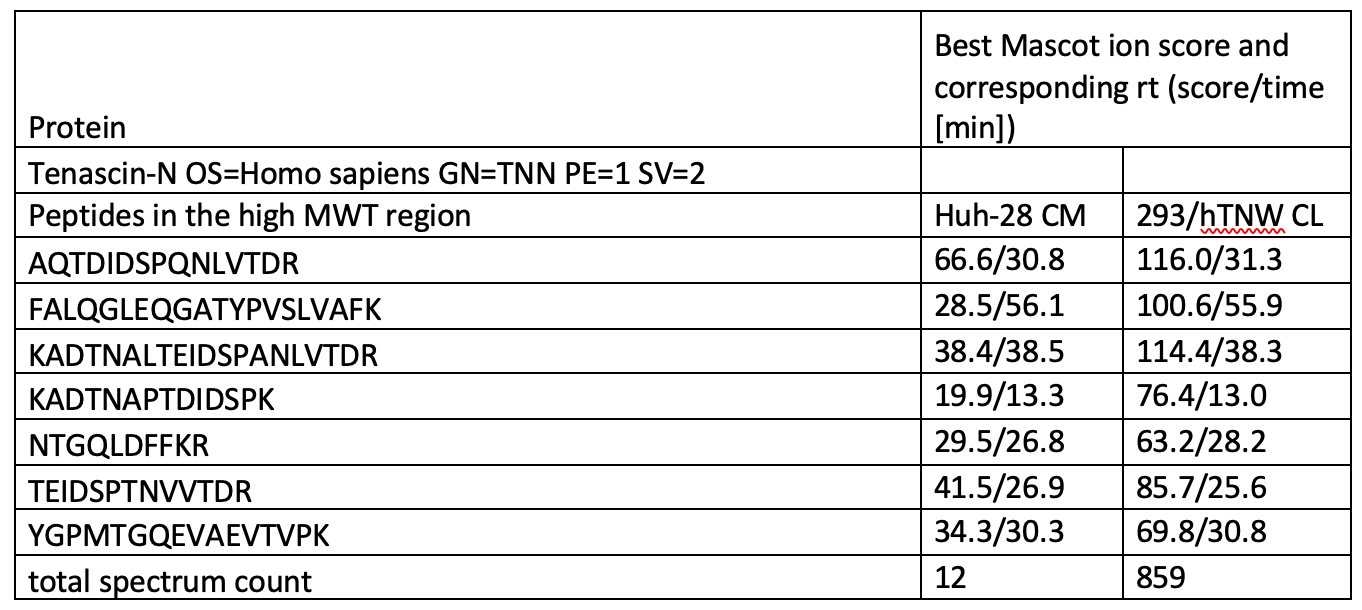
**

**B**


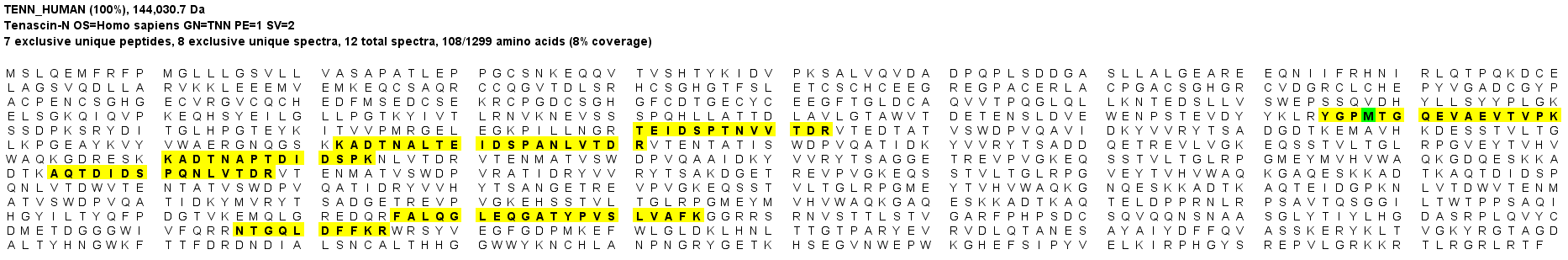


**Supplementary Figure 5** (**A**) High molecular weight (HMW) bands cut from an SDS-PAGE gel loaded with 50 µl of Huh-28 conditioned medium (CM) concentrated 30X with TCA and 250 µg of cell lysates (CL) from a control 293 cell line stably transfected with human tenascin-W (293/hTNW) were subjected to mass spectroscopy. Best Mascot ion scores and the corresponding retention time (rt) for the 7 unique tenascin-W (also called tenascin-N) peptides found in Huh-28 CM are shown. Taken together with the antibody recognition and the reduction of expression following transfection with tenascin-W-specific siRNA (Supplementary Figure 4), this is evidence that tenascin-W from Huh-28 cells runs at a higher apparent molecular weight (between 250 and 460 kDa, possibly a dimer or trimer) when analyzed with SDS-PAGE than recombinant human tenascin-W expressed by 293/hTNW cells (160 kDa). Note that the HMW bands cut from the lane loaded with Huh-28 CL were used for a pilot study using different conditions and results of their analyses are not included in this table. (**B**) Localization of the 7 unique tenascin-W peptides found jn Huh-28 CM (yellow) within the tenascin-W protein sequence.


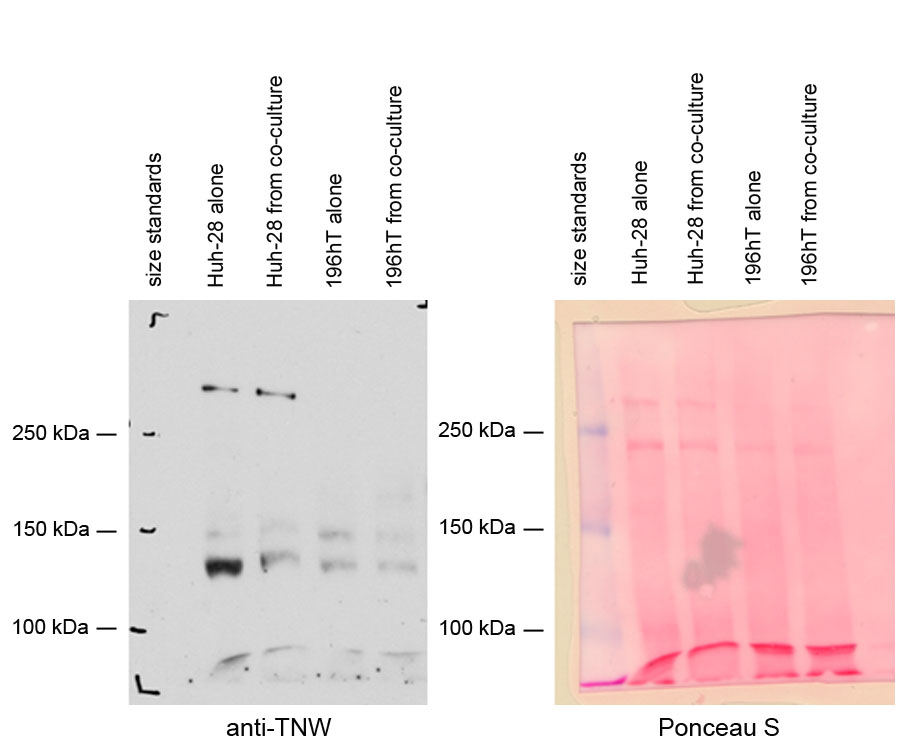


**Supplementary Figure 6** The complete immunoblot and membrane stained with Ponceau S following transfer shown as cropped images in Figure 4D (30 μg protein/lane). The high molecular weight bands (> 250 kDa) represent tenascin-W (see Supplementary Figures 4 and 5). The lower bands are artifact.


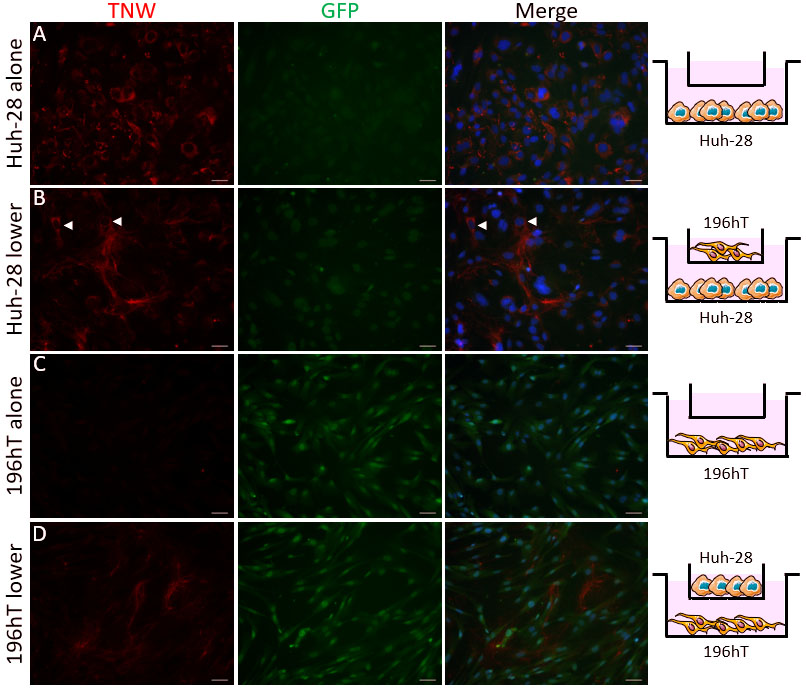


**Supplementary Figure 7**  The appearance of tenascin-W (TNW)-positive fibrils in Huh-28 cultures requires soluble factors secreted by bone marrow-derived stromal cells 196hT. Huh-28 or 196hT cells were seeded in the lower chamber in transwell cultures that only allow the diffusion of soluble factors between the chambers, and were either cultured alone (A and C) or with the other cell type in the upper chamber (B and C). TNW is detected in the cytoplasm of Huh-28 cells, but not in 196hT, when cultured separately (A and C, respectively). When 196hT cells are in the upper chamber, TNW-positive fibrils are detected around Huh-28 cells in the lower chamber, in addition to the TNW cytoplasmic localization observed in Huh-28 cells (B, arrowheads). When Huh-28 are in the upper chamber, TNW-positive fibrils are seen in 196hT cells in the lower chamber, but no TNW immunostaining is detected in the cytoplasm of 196hT cells (D). The merged images include a DAPI nuclear counterstain. Scale bars = 50 μm.
