# Supplementary material for: Tenascin-W Is a Novel Stromal Marker in Biliary Tract Cancers
Source: Front Immunol. 2021 Feb 22;11:630139. doi: 10.3389/fimmu.2020.630139 (PMC7937617; doi:10.3389/fimmu.2020.630139)
Supplement: Supplementary file 2 [file Table_1.docx]

***Supplementary Material: Table 1***

**Table 1.** **Clinicopathological features and tenascin immunostaining of biopsies from patients with biliary tract cancer**

____________________________________________________________________________________________________________________________________

*ID Age Sex Pathology* Histological type Grading*** TNM† Staging† *anti-TNC*‡ *anti-TNW*‡

____________________________________________________________________________________________________________________________________

1NT 45 M Non-tumoral liver + -

1 45 M ICC adenocarcinoma G1 T1bN0M0 IB +++ ++

2 65 F ICC adenocarcinoma G2 T1bN1M1 IV +++ +++

3NT 59 M Non-tumoral liver ++ -

3 59 M ICC squamous cell carcinoma T1bN1M0 IIB +++ +

4 29 M ICC adenocarcinoma G2 T2N1M1 IV +++ +++

5NT 75 F Non-tumoral liver +++ -

5 75 F ICC adenocarcinoma G2 T1bN1M1 IV +++ +++

6 58 F ICC adenocarcinoma G1 T1aN1M1 IV +++ +++

7 72 F ICC adenocarcinoma G2 IV +++ +++

8 59 M ICC adenosquamous carcinoma T2N2M0 IV +++ ++

9 57 M CPHBD adenocarcinoma G1 T2bN1M1 IVB +++ +

10 52 M CPHBD adenocarcinoma G1 T3bN1M1 IVB +++ +

11 51 F CPHBD adenocarcinoma G2 T1bN1M0 IIIC +++ ++

12 47 F CPHBD adenocarcinoma G1 T2bN1M0 IIIC +++ +++

13 54 M CPHBD adenocarcinoma G1 T2bN1M0 IIIC +++ +++

14 57 F CPHBD adenocarcinoma G1 T4N1M0 IIIC +++ +++

15 66 M CPHBD adenocarcinoma G1 T2bN1M0 IIIC ++ ++

16 61 M CGB adenocarcinoma G2 T3N1M1 IVB +++ +++

17 52 M CGB adenocarcinoma G2 T3N1M1 IVB +++ +++

**Table 1 (cont.).** **Clinicopathological features and tenascin immunostaining of biopsies from patients with biliary tract cancer**

____________________________________________________________________________________________________________________________________

*ID Age Sex Pathology* Histological type Grading*** TNM† Staging† *anti-TNC*‡ *anti-TNW*‡

____________________________________________________________________________________________________________________________________

18 79 F CGB adenocarcinoma G3 T3N1M0 IIIB +++ +++

19 59 M CGB squamous cell carcinoma pT1bN0M0 IA +++ +++

20 79 F CGB adenocarcinoma G3 T3N2M0 IIIB +++ +++

21 66 F CGB adenocarcinoma G2 T3N1M0 IIIB +++ +

22 72 F CGB mucinous carcinoma T3N1M0 IIIB +++ +++

23 73 M CGB adenocarcinoma G1 T3N1M1 IVB +++ ++

24 60 M CGB adenocarcinoma G3 T3N1M0 IVB +++ +++

25 63 F CGB adenocarcinoma G2 TSN1M1 IVB +++ ++

26 70 F CGB adenocarcinoma G1 T3N1M1 IVB +++ ++

27 59 M CGB liver metastasis adenocarcinoma G2 T2N2M1 IVB +++ +++

28 67 F CGB liver metastasis adenocarcinoma G2 T3NxM1 IVB +++ ++

29 46 M CGB liver metastasis adenocarcinoma G2 T3N2M1 IVB +++ ++

30 62 F CGB liver metastasis adenocarcinoma G1 T3N2M1 IVB +++ +++

____________________________________________________________________________________________________________________________________

*ICC, intrahepatic cholangiocarcinoma (ICD-O-3 C22.1); CPHBD, carcinoma of perihilar bile ducts (ICD-O-3 C24.0); CGB, carcinoma of the gallbladder (ICD-O-3  C23.0)

**G1, well differentiated; G2, moderately differentiated; G3, poorly differentiated

†Union for International Cancer Control. TNM Classification of Malignant Tumors, 8^th^ Ed.

‡Intensity of anti-tenascin-C (TNC) and anti-tenascin-W (TNW) immunostaining: -, absent; +, weak; ++, moderate; +++, high
